# Nutritional Status and Cardiovascular Health in Female Adolescent Elite-Level Artistic Gymnasts and Swimmers: A Cross-Sectional Study of 31 Athletes

**DOI:** 10.1155/2021/8810548

**Published:** 2021-01-12

**Authors:** Boštjan Jakše, Barbara Jakše, Nataša Fidler Mis, Borut Jug, Dorica Šajber, Uroš Godnov, Ivan Čuk

**Affiliations:** ^1^Department of Food Science, Biotechnical Faculty, University of Ljubljana, Ljubljana, Slovenia; ^2^Barbara Jakše Sole Proprietor, Domžale, Slovenia; ^3^Department of Gastroenterology, Hepatology and Nutrition, University Children's Hospital, University Medical Centre Ljubljana, Ljubljana, Slovenia; ^4^Centre for Preventive Cardiology, Department of Vascular Diseases, University Medical Centre Ljubljana, Ljubljana, Slovenia; ^5^Faculty of Medicine, University of Ljubljana, Ljubljana, Slovenia; ^6^Department of Swimming, Faculty of Sport, University of Ljubljana, Ljubljana, Slovenia; ^7^Department of Computer Science, Faculty of Mathematics, Natural Sciences and Information Technologies, University of Primorska, Koper, Slovenia; ^8^Department of Gymnastics, Faculty of Sport, University of Ljubljana, Ljubljana, Slovenia

## Abstract

**Objective:**

Nutritional status is important for health and competitive achievement. This area remains understudied among elite-level female athletes and is appropriate for research. We examined nutritional status and cardiovascular health markers of two groups of female athletes of the same age and competition period, involved in weight-bearing and a non-weight-bearing sport: gymnasts (*n* = 17) and swimmers (*n* = 14); mean age 17.4 and 16.6 years.

**Methods:**

Body composition and dietary intake were assessed by bioelectrical impedance and Food Frequency Questionnaire. The concentrations of serum micronutrients (B_12_, 25-hydroxyvitamin *D* (25 (OH) D), calcium, magnesium, phosphorus, potassium, and iron), blood lipids, and blood pressure (BP) were measured. *Setting and Participants*. A cross-sectional study of 31 athletes from Slovenia.

**Results:**

Gymnasts had higher body mass index (21.5 vs. 20.1 kg/m^2^, *p*=0.043) and lower fat free mass (42.4 vs. 46.6 kg, *p*=0.024) than swimmers and comparable body fat percentage (22.5 vs. 22.8%). Both groups had low intake of carbohydrates, fibre, polyunsaturated fats, protein (only gymnasts), and micronutrients (11/13 micronutrients gymnasts and 4/13 swimmers) and high intake of free sugars and saturated fats. Both groups also had significantly lower-than-recommended serum levels of 25 (OH) D. All cardiovascular risk factors were within recommended ranges. Gymnasts had higher LDL cholesterol (2.7 vs. 2.2 mmol/L, *p* < 0.011), and swimmers had higher systolic BP (126 vs. 107 mmHg, *p* < 0.001).

**Conclusions:**

Dietary intake especially in gymnasts was suboptimal, which may reflect in anthropometric and cardiovascular marker differences between gymnasts and swimmers.

## 1. Introduction

Body mass management of elite athletes is pivotal for sport participation and competitive achievement, especially in sports where the athlete's body mass is an important determinant of success, such as artistic gymnastics or swimming [1, 2]. Appropriate nutrition is essential for athletic performance and health maintenance [3], but data on dietary intake of highly competitive athletes are limited. Dietary intake of athletes, including elite female artistic gymnasts and swimmers, is often assumed to be nutritionally adequate, but current evidence suggests that assumption may not always be the case [4–6]. Although optimal diet and a healthy lifestyle are the main prerequisites for sports participation, even well-trained athletes are prone to developing diseases as they age [7, 8].

Artistic gymnasts may be especially at risk because of specific dietary patterns and needs [9, 10] due to high training volume [11] and low body fat requirement that is further important contributor to success in artistic gymnastics [12]. Previous research frequently emphasizes that the dietary habits of female gymnasts may be suboptimal [4, 5]. It seems that the dietary intake of female artistic gymnasts is nutritionally inadequate and may often border on eating disorders [13]. The risk of suboptimal energy intake is higher in aesthetic sports, such as gymnastics, and may produce an increased risk for injuries and decreased athletic performance [14]. Several studies have confirmed serum deficiencies in 25-hydroxyvitamin *D* (25 (OH) D) [6, 15], which may increase the risk of stress fractures and illness, increase muscle weakness, and delay muscle recovery [16].

Dietary requirements for swimmers are dependent on swimming style (i.e., freestyle, breaststroke, backstroke, or butterfly), competitive distances (e.g., 50–800 metres), training requirements, and competition phase [17, 18]. A study of dietary intake in adolescent swimmers identified low intakes of several nutrients, especially calcium and vitamin D and high intakes of total fat and saturated fat, which may adversely impact performance and increase the risk for chronic diseases [19]. Several other studies, especially among female swimmers, reported low energy intake and suboptimal nutrient status (e.g., calcium, iron, and vitamin D) [20].

A healthy diet has been the cornerstone of cardiovascular diseases (CVDs) prevention and treatment for decades [21]. Specifically, saturated fatty acids (SFAs) and refined carbohydrates have been associated with an increased risk for CVD [21, 22]. Hence, dietary patterns for the reduction in CVD risk emphasize high intake of fruits, vegetables, whole grains, and legumes, moderate intake of nuts, and limited quantities of lean meat (including poultry and seafood), low-fat dairy products, and liquid vegetable oils. Overall, the recommended dietary patterns are all low in transfatty acids and SFA, sodium, free sugars, and ultraprocessed foods, including refined grains [21, 23].

Athletes may have an increased risk of CVD later in life, although data on the association between elite sports participation and cardiovascular health remains largely inconclusive. Studies in endurance runners and team sport athletes, for instance, have demonstrated a relatively high incidence of CVD later in life [7, 8]. However, a cross-sectional study on former elite football players showed a reduced cardiac risk factors compared to never athletic men matched for age later in middle age, when the athletes remained physically active [24]. Importantly, in another cross-sectional study with follow-up of 21.3 ± 2.8 months on 108 apparently healthy male marathon runners (age ≥ 50 years), there were no differences in the prevalence of coronary artery calcification (CAC) compared to age-matched controls (i.e., meaning moderate plaque deposits) present in 36% of runners [25]. At present, however, it is not clear whether this tendency reflects specific dietary patterns and cardiometabolic dysfunction, long-term sport activity-associated cardiovascular (mal) adaptations, or both.

In terms of possible cardiometabolic dysfunction, several humoral markers have been associated with increased morbidity and mortality in the general population and in athletes. Blood lipid levels (especially low-density lipoprotein cholesterol (LDL cholesterol) and triglycerides) and blood pressure (BP) represent important determinants of cardiovascular health [26, 27]. There is also an established relationship between serum uric acid (S-UA) levels and the risk of CVD mortality [28]. The relationship, however, seems U-shaped. While very low S-UA levels have been associated with neurodegenerative diseases (such as multiple sclerosis, Alzheimer's disease, and Parkinson's disease), cancer, and vascular disease-related dementia, hyperuricaemia has been associated with chronic kidney disease, CVD, type 2 diabetes, and dyslipidaemia [29]. Impaired fasting serum glucose in the nondiabetic range has also been associated with subclinical CVD [30]. Last, haemoglobin levels, either below or above the reference range, have recently been associated with increased CVD and all-cause mortality [31]. In the assessment of the pathophysiological relationship between red blood cell disorder and CVD, evaluation of iron and vitamin B_12_ status remain pivotal [32].

There is a lack of studies examining a nutritional and health status in elite-level female athletes. The present study is a component of a larger cross-sectional study on various aspects of elite-level female indoor athletes, who were gymnasts and swimmers matched according to age and competition period. The study examined athletes involved in a weight-bearing (i.e., aesthetic group; aesthetic components are judged) and a non-weight-bearing (i.e., non-aesthetic group; aesthetic components are not judged) sport. Both groups of athletes are faced with the problem of a predominantly indoor institutionalized lifestyle and have special dietary requirements [9, 18]. In the previous paper on these participants, we demonstrated that bone mineral density between these two groups differs despite no detectable differences in serum 25 (OH) D or calcium concentrations (S–Ca) [33].

In the present study, we aimed to investigate the anthropometric measures, dietary intake, serum micronutrient concentrations, and cardiovascular health status of two elite-level indoor female athlete groups.

## 2. Materials and Methods

### 2.1. Study Design and Eligibility

The study was conducted during the competition period and within three days (i.e., between April 4^th^ to 6^th^ 2018) to mitigate possible seasonal effects and training status. Blood samples were collected and measurements performed after an overnight fast. For complete biochemical assays (e.g., serum micronutrients and CVD risk assay), a single sample of 15 mL of blood was taken. The gymnasts were tested at Ljubljana Medical Centre (Ljubljana, Slovenia), whereas the swimmers were tested at Maribor Medical Centre (Maribor, Slovenia).

This cross-sectional study was approved by the national Medical Ethics Committee of the Republic of Slovenia (No. 0120–177/2018) and registered at https://clinicaltrials.gov (NCT03584256). All participants and/or their parents/legal guardians (for participants below the age of 18) signed an informed consent at inclusion. The study was conducted in accordance with the Declaration of Helsinki. Participants were not remunerated financially for participation in the study.

### 2.2. Subjects

We included 31 elite-level female indoor athletes: 17 gymnasts (aged 17.4 ± 4.1 years) and 14 swimmers (aged 16.6 ± 3.1 years). All participants were from the same ethnic group (Caucasian), resided in Slovenia, and many of them (58%) were performing at the highest international competitive level (e.g., European Championships, World Cups, and Olympic Games). The participants were invited through personal contact with the National team coach and other gymnastic and swimming coaches working in clubs in Slovenia. The criteria for inclusion in the study were competing internationally and nationally, currently fully actively involved in a training program, and no use of any prescribed medications that affect bone metabolism. All invited athletes who met the inclusion criteria responded to the invitation, and we did not exclude anyone's data from the final analysis. All measurements (e.g., anthropometrics, blood assays, and blood pressure) that were part of the study were paid for by the Slovenian Research Agency.

### 2.3. Outcomes

The outcome variables included basic characteristics (e.g., training volume, menarche status, and dietary pattern), basic anthropometric measures, dietary intake, serum micronutrient concentrations, and CVD risk and safety factors.

#### 2.3.1. Basic Characteristics and Anthropometric Measures

The amount of training and menarche data were evaluated by a questionnaire developed by the authors that included questions about hours of training each day of the week, year of first menstrual period, regular/irregular menstrual cycle, and type of menstrual period (painful/not, heavy/light).

Basic anthropometrics included body height, body mass, and body mass index (BMI), body fat percentage (BF%), and fat free mass (FFM). Heights (cm) were measured by the body height gauge (Kern, MPE 250K100HM, Kern and Sohn, Balingen, Germany). Fat free mass (FFM) and BF% was assessed by an 8-electrode medically approved and calibrated bioelectrical impedance body composition monitor (Tanita 780 S MA, Tanita Corporation, Tokyo, Japan), which provides an valid tool to measure total BF% in healthy young males and females regardless of their level of habitual physical activity [34]. Accuracy of FFM and BF% are 0.1 kg and 0.1%. Body composition was measured after overnight fasting and before blood sample collection. Participants were measured according to manufacturer's recommendations [35]. Before the bioimpedance measurement, participants were also asked not to drink for at least one hour, not to exercise for at least 24 h (the measurement took place on the second day of recovery), or urinate for at least 30 minutes. Females were not measured three days before or after menstruation. All measurements were carried out by the same researcher (first author) who was appropriately trained. Dual-energy X-ray absorptiometry (DXA) was used to measure bone mineral density, and the results are published in another paper [33].

#### 2.3.2. Dietary Intake

To assess dietary habits in the past year, we used a manual monitoring technique, a 52-item qualitative Food Frequency Questionnaire (FFQ), that was based on a validated 50-item FFQ [36]. The FFQ had been translated from the original Dutch language into the Slovenian language by a professional translator. This FFQ included nine different frequency categories ranging from ‘never' to ‘more than 3 times a day'. The usual food intakes derived from the FFQ were calculated by multiplying the frequency of consumption of specific foods by a standard portion size for each food (as proposed by National Institute of Public Health of Slovenia, [37]) and by the amount of nutrient present in one gram. The daily dietary intake was calculated by summing the nutrient content of each food item. The FFQ has been tested and validated for assessing food consumption with 7-day estimated diet records [38]. To calculate daily dietary intake, we used Dietplan7 Pro dietary assessment software (Forestield Software Limited, Horsham, UK) [39]. Dietplan7 Pro is a comprehensive, high-quality nutrition analysis software package for professionals that uses live database from several suppliers tables and national food tables [39]. We have used the manual method and double checked it to avoid any possible errors in such a small sample. All dietary supplements and sport drinks were included in the evaluation of dietary intake. Additionally, we included information on dietary supplements and sport drink intake in the descriptive analysis (see 3.2.3).

We calculated data for dietary intake of gymnasts and swimmers expressed as kcal/day (energy), g/day (macronutrients), except for dietary cholesterol (mg/day) and water intake (L/day), % of daily energy intake (macronutrients), kcal/kg FFM/day (energy intake), and g, mg, or *μ*g of macro- and micronutrients/kg BM/day (unit of nutrient/kg BM/day).

The energy availability (EA) and several macronutrients intakes (i.e., carbohydrates, total fat, and total protein) were compared with the joint position of the Academy of Nutrition and Dietetics, Dietitians of Canada, and the American College of Sports Medicine for nutrition and athletic performance [40], whereas the intakes of fibre, SFA, mono- and polyunsaturated fatty acids (MUFA and PUFA), fat, cholesterol, and micronutrients were compared with the Central European (German (D), Austrian (A), and Swiss (CH) D-A-CH) reference values for the 15 to < 19 years age group [41]. D-A-CH references values are valid in Slovenia [42]. The zinc intake recommendation in our study was based on results of reported intake of food groups and used for diets with low intake of whole grains and legumes and high intakes of animal protein [42]. The intake of free sugars, as defined by the World Health Organization [43], was compared with recommendations from UK Scientific Advisory Committee on Nutrition (SACN) [44] and European Society for Paediatric Gastroenterology, Hepatology and Nutrition (ESPGAHN) [45] of <5% of daily energy intake. Finally, we evaluated water intake from solid foods and beverages. We did not compare total water intake with the guidelines since total water intake depends on the sport, the type of exercise, and the environment [40].

#### 2.3.3. Serum Micronutrient Concentrations

Serum micronutrients that are frequently of concern were assessed in a certified biomedical laboratory in Ljubljana (gymnasts) and Maribor (swimmers) and included analysis of vitamins B_12_ (S-vit B_12_) and *D* (measured as the 25 (OH) D, calcium (S–Ca), magnesium (S–Mg), phosphorus (S–P), and potassium (S–K), and trace element iron (S–Fe). For serum micronutrients, both laboratories (for gymnasts and swimmers) used the same manufacturer and methodology.

The S–B_12_ levels of all the participants were measured using a competitive-binding immunoenzymatic assay, the Elecsys Vitamin B_12_ II assay (ECLIA), according to the manufacturer's instructions (Roche Elecsys 2010). The assay has a linearity range of 36.9–1.476 pmol/L. For the S-vit B_12,_ level, there is no clear, accepted cutoff value to define deficiency, but for additional comparison of athlete micronutrient status and recommended values, the S-vit B_12_ was compared with 258 pmol/L as suggested by German researchers to prevent neurocognitive disorders late in life [46].

The 25 (OH)D levels were measured using a commercially available Elecsys® Vitamin D total assay with a Cobas e601 analyser (Roche Diagnostics International Ltd., Rotkreuz, Switzerland), which uses a competitive electrochemiluminescence binding technique. The method was standardized according to internationally accepted procedures. For the purpose of interpreting 25 (OH) D levels, which is a known challenge for indoor athletes [47], we used three categories (e.g., 25 (OH) D sufficiency: >75 nmol/L, insufficiency: 50–≤75 nmol/L, and deficiency: <50 nmol/L) [48].

The S–Ca concentrations were determined on an automated clinical chemistry analyser (Beckman Coulter AU 640, AU, Austria) device using colorimetric photometric methods based on the Arsenazo III complex. For total Ca, the reaction between Ca^2+^ in the sample and Arsenazo III results in a purple complex, the absorbance is measured bichromatically at 660/700 nm, and the increase in absorbance is directly proportional to the total calcium concentration in the sample.

The S–Mg concentrations were determined on an automated clinical chemistry analyser (Beckman Coulter AU 640, AU, Austria) by a photometric colour test for the quantitative determination of magnesium in human serum. The magnesium reagent utilises a direct method in which magnesium ions form a coloured complex with xylidyl blue in a strongly basic solution. The colour produced is measured bichromatically at 520/800 nm and is proportional to the magnesium concentration in the serum.

The S–P concentrations were determined on an automated clinical chemistry analyser (Beckman Coulter AU 640, AU, Austria) that uses System Calibrator for serum application. Inorganic phosphorous reacts with molybdate to form a heteropolyacid complex. The use of a surfactant eliminates the need to prepare a protein-free filtrate. The absorbance at 340/380 nm is directly proportional to the inorganic phosphorous concentration in the sample.

The S–K concentrations were determined on an automated clinical chemistry analyser (Beckman Coulter D × C 800, AU, Austria) using a precise volume of sample (40 microliters) that was mixed with a buffered solution (ISE Electrolyte Reference reagent). The ratio used was one-part sample to 33 parts buffer. The high molar strength buffer is used to establish a constant activity coefficient for potassium ions, calibrating the electrode to concentrations.

The S-Fe concentration was determined using a Vitros 950 analyser (Ortho-Clinical Diagnostic, Rochester, N. Y., USA). S-Ca concentrations were determined using a Beckman Coulter AU 640 (Beckman Coulter AU, Austria), and S-P concentrations were determined on an automated clinical chemistry analyser (Beckman Coulter AU 680, Austria).

Serum mineral concentrations of gymnasts and swimmers were compared with the reference values of the University Medical Centre Ljubljana, Slovenia, which is the national core laboratory [49].

#### 2.3.4. Cardiovascular Disease (CVD) Risk and Safety Factors

Appraised CVD risk factors were total cholesterol (S-cholesterol), high-density lipoprotein (HDL cholesterol) and LDL cholesterol measured directly, and blood pressure (BP). The safety markers that we included in the blood analysis were S-UA, fasting glucose (S-glucose), and haemoglobin.

For biochemical analyses of lipids, S-glucose, and S-UA, a Beckman Coulter AU 640 (Beckman Coulter, Austria) was used at one laboratory (for gymnasts), whereas a Becton Dickinson Vacutainer® SSTII Advance (BD, Plymouth, UK) analyser was used at another laboratory (for swimmers). For serum haemoglobin concentrations, both laboratories used an Advia 2120i (Siemens, Healthcare Diagnostics, Germany) haematology analyser. Blood pressure (BP) was assessed using an oscillometric technique in the supine position after five minutes of rest and before taking the blood sample. The average of two measurements three minutes apart was used for analysis. LDL cholesterol and BP were our primary CVD risk factors.

To assess the proportion of participants reaching the recommended targets for LDL cholesterol (<3.4 mmol/L) and triglycerides (<1.7 mmol/L), we used the values from the European Atherosclerosis Society (EAS) [50]. Furthermore, to offer detailed interpretation of our findings, we used an additional threshold for elevated LDL cholesterol (i.e., ≥2.6 mmol/L). LDL cholesterol ≥2.6 mmol/L is considered to be associated with subclinical atherosclerosis, even in the absence of other risk factors [51, 52], which may become a serious health concern or even fatal for athletes later in life. For BP ≤ 129/84 mmHg, we used the recommendation of the European Society of Cardiology (ESC) [53]. For S-cholesterol and HDL cholesterol values for gymnasts and swimmers, we used the reference values from the University Medical Centre Ljubljana, Slovenia, which is the national core laboratory [49].

There is no clear consensus for normal S-UA. However, a threshold value < 360 *μ*mol/L seems to identify true healthy subjects for all subjects [54]. For S-glucose (<5.8 mmol/L), we used the recommendation from the European Diabetes Epidemiology Group for lean adults (BMI < 25 kg/m2) [55]. For haemoglobin, we used a recommended cutoff for a nonanaemic state from the World Health Organization (WHO) for nonpregnant females (>120 g/L) [56].

### 2.4. Statistical Analysis

Statistical analysis was performed with R 3.5.2 with the dplyr [57], ggplot2 [58], and arsenal [59] packages. Dplyr was used for data transformation, ggplot2 for data visualization, and arsenal for statistical calculations. dplyr data were not rescaled (i.e., log operations) but just transformed from a wider format to longer one (de-pivoting), calculating percentages and totals. The number of elite-level athletes is extremely small; therefore, we were targeting all artistic gymnasts and swimmers that met our inclusion criteria. All the gymnasts and swimmers who were invited and met the criteria accepted our invitation, so calculating the sample size would not be relevant. For comparing data between gymnasts and swimmers, we used the Mann–Whitney *U* test for independent samples, and for comparing intakes against recommendations, we used one-sample Wilcoxon signed-rank test exclusively. We chose the nonparametric tests because the sample size was quite small, and the skewness coefficient indicated that all variables, except from three, were not normally distributed. Further exploration of remaining three variables showed the differences between the “normality” in groups. Importantly, where the dietary intake recommendations were specified as a range (i.e., such as for carbohydrates, total fat or PUFA intake), we took the average of the range. The threshold for statistical significance was less than 0.05. No missing data were present. No sensitivity analysis was performed. Data are presented as the means (standard deviation).

## 3. Results

### 3.1. Basic Characteristics and Anthropometric Measures

Characteristics of gymnasts and swimmers are presented in Table 1. The gymnasts started with regular gymnastics training at a mean 5.3 ± 2.7 years. At the time of the study, they had 23.5 ± 3.4 hours of training weekly. They had all been involved in an average of 10.5 ± 3.4 years of systematic training. However, the swimmers started with regular swimming training at a mean age of 9.3 ± 1.9 years. At the time of the study, they completed 45.4 ± 3.5 km swimming weekly. They had all been involved in training with an average of 8.5 ± 3.5 years of systematic training. Most swimmers (86%) were specialized for shorter competition disciplines (50–200 metres).

53% of gymnasts and 86% of swimmers reported consuming an omnivorous diet, whereas others characterized their diet as vegetarian or occasionally vegetarian. Important, 18% of gymnasts and 21% of swimmers reported to have irregular menstrual cycle.

### 3.2. Dietary Intake Status

**Figure 1:**
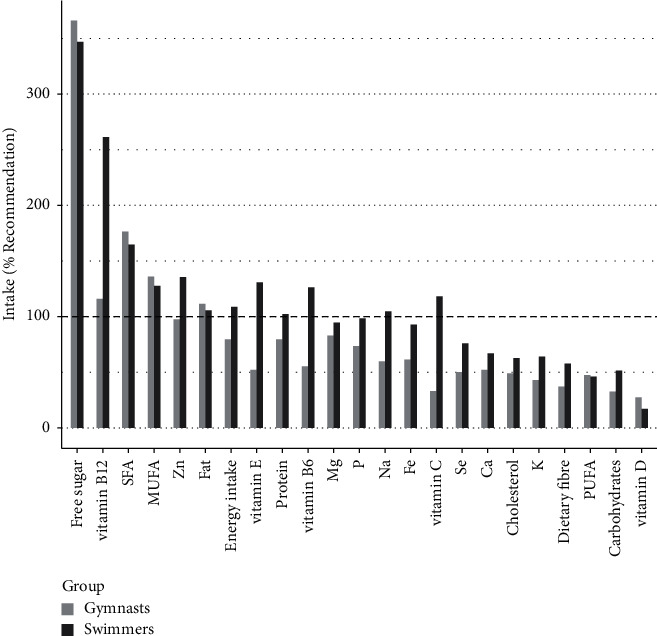
Dietary intake of elite-level artistic gymnasts and swimmers compared with recommendations [40–45].

Comparisons between daily energy and nutrient intakes of gymnasts and swimmers and comparisons with recommendations are presented in Table 1S, Table 2, and Figure 1, whereas relative dietary intakes expressed per kcal/kg FFM/day (energy intake) and per unit of nutrient/kg BM/day are presented in Table 2S.

#### 3.2.1. Energy and Nutrient Intake

The reported absolute energy intake was significantly lower in gymnasts than in swimmers (mean ± SD) (1514 ± 258 vs. 2263 ± 407 kcal/day) (*p* < 0.001). The mean proportions of ingested energy among the main macronutrients for gymnasts were 47% from carbohydrates, 40% from fat, and 14% from protein, whereas in swimmers, it was 54% from carbohydrates, 38% from fat, and 13% from protein. Water intake from beverages and solid foods was higher in swimmers than in gymnasts (2.54 ± 0.27 vs. 1.97 ± 0.21 L/day,*p* < 0.001). Between the studied groups, there were no significant differences in intake of macronutrients (e.g., for free sugars, total fat, saturated fatty acids or protein, expressed as % of daily energy intake, and for cholesterol expressed as mg/day). Furthermore, there were no significant differences in intakes of several micronutrients (e.g., vitamin D, calcium, and magnesium).

The EA was significantly lower than recommended in both groups, but more in gymnasts than in swimmers (23 ± 3 and 33 ± 5 kcal/kg FFM/day in gymnasts and swimmers vs. recommended 45 kcal/kg FFM/day; *p* < 0.001 in gymnasts and *p* < 0.05 in swimmers). Gymnasts and swimmers consumed the daily recommended intake (% of daily energy intake) of fat and MUFA, and swimmers also consumed the daily recommended intake of protein, whereas both groups consumed significantly below reference intakes of carbohydrate (kcal/kg FFM/day), fibre (g/day), and PUFA (% of daily energy intake). Both groups consumed high amounts (i.e., higher than the upper limit) of free sugars and SFA but acceptable amounts of dietary cholesterol (the recommended < 300 mg/day). Concerning the intake of micronutrients, gymnasts met the recommended intake for only two (2/13) (vitamin B_12_ and zinc), whereas swimmers met recommendations for nine micronutrients (9/13).

#### 3.2.2. Intake of Food Groups and Enriched Foods/Beverages (Such as Sport Drinks)

Fruits (including in the form of a smoothie or as dry fruits) and vegetables (in cooked or fresh) were not consumed by all gymnasts or swimmers, whereas no one in either group reported regular daily consumption of legumes, potatoes, plant-based meat alternatives (i.e., tofu, tempeh, and seitan), or fish.

Specifically, 29% of gymnasts and 21% of swimmers consumed fruits at least once per day, whereas 47% of gymnasts and 50% of swimmers consumed fruits 2-3 times per day. Twenty-four percent of gymnasts and 29% of swimmers did not consume fruits on a regular basis. Twenty-nine percent of gymnasts and 14% of swimmers consumed vegetables (cooked or fresh) at least once per day, whereas no gymnasts and 7% of swimmers consumed vegetables 2–3 times per day. Whole grains were consumed at least once per day by 41% of gymnasts and 64% of swimmers, whereas 12% of gymnasts and 36% of swimmers consumed whole grains 2–3 times per day. Forty percent of gymnasts and 36% of swimmers reported consuming whole grain foods 2–4 times weekly. Only 8% of gymnasts and none of the swimmers consumed nuts and seeds daily, whereas 30% of gymnasts and 43% of swimmers did not consume any nuts or seeds.

Dairy and meat products were consumed daily by 41% and 12% of gymnasts and 29% and 50% of swimmers, respectively, whereas eggs were consumed daily by 6% of gymnasts and 7% of swimmers. Only 23% of gymnasts and 21% of swimmers consumed fish at least once weekly.

Processed plant-based food products (e.g., fruit juice, sweets, white flour products, and marmalade) were reportedly consumed daily by 65% of gymnasts and 79% of swimmers, whereas processed meat (e.g., sausage and salami) or ultraprocessed foods (mayonnaise, margarine, butter, lard, and fried foods) were reportedly consumed very rarely by gymnasts (18% of them 2–4 times weekly) and more frequently by swimmers (43% of them 2–4 times weekly).

A total of 88% of gymnasts and 86% of swimmers drank water more than 3 times/day, whereas other drinks that were reported to be consumed for both groups were coffee or tea. Sport drinks were consumed by the majority of swimmers (71%) but only a minority of gymnasts (6%). Fifty-seven percent of swimmers consumed sport drinks two or three times per day (during training). Gymnasts and swimmers reported not consuming any alcohol on a daily or weekly basis.

#### 3.2.3. Intake of Dietary Supplements

Dietary supplements or sport drinks were not consumed by 41% of gymnasts and 14% of swimmers. The most frequently consumed dietary supplement among gymnasts was magnesium (35%), whereas swimmers consumed multivitamins (21%). Eighteen percent of gymnasts and no swimmers consumed vitamin *D* supplements. Eighteen percent of gymnasts and 7% of swimmers consumed dietary supplements of omega-3 long-chain PUFA (eicosapentaenoic acid (EPA) and docosahexaenoic acid (DHA)). Twelve percent of gymnasts consumed calcium, and 12% of gymnasts consumed iron supplements, whereas no swimmers consumed calcium and 7% of swimmers consumed iron supplements. Among swimmers, 14% consumed dairy protein shakes (whey protein) compared to 6% of gymnasts.

#### 3.2.4. Meal Frequency

Most gymnasts and swimmers (59 and 64%) regularly consumed all three main meals: breakfast, lunch, and dinner. Six percent of gymnasts and 7% of swimmers reported never eating breakfast, whereas 29% of gymnasts and 14% of swimmers reported not consuming breakfast every day. Twenty-three percent of gymnasts and 29% of swimmers did not eat lunch every day, and 18% of gymnasts and 36% of swimmers skipped dinner one-two times per week.

### 3.3. Serum Micronutrient Status

Serum micronutrients are presented in Table 3. Gymnasts had significantly higher S-K and S-P status compared to swimmers. Others serum micronutrients were not significantly different between the groups.

All mean serum micronutrients for both athlete groups were within the recommended range, except for 25 (OH) D for both groups and S–P for gymnasts. The mean 25 (OH) D levels were sufficient (>75 nmol/L) in 23% of gymnasts and in 43% of swimmers, insufficient (50–74 nmol/L) in 35% of gymnasts and 36% of swimmers, and deficient (<50 nmol/L) in 42% of gymnasts and 21% of swimmers.

Other minerals that were also not within reference ranges for all gymnasts were S–Fe and S–P, whereas for swimmers, S-P and S-Mg were not in reference ranges. S-Fe was found not to be within the reference range for 23% of gymnasts, whereas S-P was found to be higher than the upper threshold of the reference range for 35% of the gymnasts and 21% of swimmers. S-Mg was found to be within the reference range for all gymnasts and swimmers. The mean value of S-vit B_12_ was found to be, on average, above the reference values for gymnasts and swimmers. However, only 53% of gymnasts and 79% of swimmers had S-vit B_12_ above the reference value of 258 pmol/L.

### 3.4. Cardiovascular (CV) Health and Safety Factors

Cardiovascular health (CV) status is shown in Table 4. Gymnasts had significantly higher LDL cholesterol compared with swimmers, whereas swimmers had significantly higher HDL cholesterol, S-glucose, S-UA, and systolic BP. No differences were detected when comparing S-cholesterol, triglycerides, or diastolic BP.

All blood variables and BP were, on average for both groups, within recommended target ranges. However, there are some important insights within groups that help interpret the athletes' CV status. Ninety-four percent of the gymnasts and 93% of the swimmers had S-cholesterol values within the reference range, whereas 71% of gymnasts and 93% of swimmers had LDL cholesterol within recommendations. Furthermore, 59% of the gymnasts had LDL cholesterol above 2.6 mmol/L, and 41.2% had LDL cholesterol above 3 mmol/L, whereas these numbers among swimmers were lower (21% swimmers had above 2.6 mmol/L and 7% above 3 mmol/L). HDL cholesterol values were within recommendations in 82% of gymnasts and 100% of swimmers. Furthermore, 35% of gymnasts and 29% of swimmers had triglycerides ≤0.6 mmol/L. None of the athletes had triglycerides above the reference value. BP was below the recommended target (≤129/84 mmHg) for all gymnasts, whereas systolic BP and diastolic BP were higher than recommended in 36% and 7% of swimmers, respectively.

Importantly, none of the gymnasts and swimmers had S-glucose outside the recommended level (<5.8 mmol/L). Furthermore, all gymnasts and swimmers had S-UA values below the threshold value (<360 *μ*mol/L). One gymnast and no swimmers had haemoglobin values below the recommended cutoff (≥120 g/L).

## 4. Discussion

### 4.1. Main Findings

First, our results confirm diverse body compositions among elite-level female gymnasts and swimmers. Gymnasts were shorter and lighter, but they had a higher BMI and lower FFM. There were no discernible differences between gymnasts and swimmers only for BF%.

Second, the intake of energy and most nutrients were lower among gymnasts than swimmers, with the exception of free sugars (g/day), SFA, protein (% of energy), and several micronutrients (e.g., vitamin D, calcium, and magnesium).

The dietary intake of gymnasts and swimmers, when compared to current recommendations and duly acknowledging the limitations of our methodology (i.e., FFQ), may be regarded as suboptimal. In particular, free sugars and SFA exceeded the upper limit of acceptable intakes and showed ample room for improvement.

Third, gymnasts had significantly higher S–K and S–P status compared with swimmers. Furthermore, serum micronutrients were on average within recommended ranges for both groups, except for 25 (OH) D for both groups and S–P for gymnasts. Further analysis, however, suggests that 25 (OH) D and S-vit B_12_ represent the most challenging micronutrients.

Finally, markers of cardiovascular health were within guidelines/recommended ranges. However, gymnasts had significantly higher LDL cholesterol compared to swimmers, whereas swimmers had significantly higher systolic BP than gymnasts. In terms of safety factors, average S-glucose, S-UA, and haemoglobin values were in line with recommendations.

### 4.2. Anthropometric Measures

Our results indicate that gymnasts, compared to swimmers, were shorter and lighter but had higher BMI, lower FFM, and comparable BF%. By comparison, a recent anthropometric study suggested a mean body height of 153 cm and mean body mass of 44 kg in elite-level female artistic gymnasts with a mean age of 15.8 years [61]. Previous research consistently indicated that leanness is an important contributor to success in artistic gymnastics. BF% measurements in most successful elite-level female gymnasts suggest very low body fat composition (ranging from 11.3 to 16%) [12]. However, our results showed evident differences in measured values than those evidenced in previous studies on samples of athletes with similar competition levels from the United States of America (e.g., US national teams) but were not age matched (17.4 vs. 15.5 vs. 15.8 years for Slovenian and two US teams) [62, 63]. More precisely, Slovenian gymnasts were taller and heavier and had a higher BMI than 42 gymnasts in first US study (body height: 159.8 cm vs. 150.9 cm, body mass: 54.8 kg vs. 46.5 kg, BMI: 21.5 kg/m^2^ vs. 20.3 kg/m^2^, for Slovenian and US gymnasts, respectively) [62]. Furthermore, when comparing our results with another US study on 48 elite-level gymnasts, these gymnasts were also significantly younger (15.8 years), shorter (152.2 cm), and lighter (47.7 kg) and had lower BF% (14.3%) but higher relative FFM (84.9 vs. 77.4% of BM) [63]. Importantly, limitations in comparing body composition may be, in apart, from different assessment technologies. The US studies of body composition used DXA, whereas we used medically approved and calibrated bioelectrical impedance. To conclude, available data in recent systematic reviews of elite-level female gymnasts used mostly outdated studies, and recent data are still not sufficient for providing robust conclusions whether body composition features actually explain competitive performance [12].

For female swimmers, previous studies yielded even more inconclusive information. One US cross-sectional study on 43 female competitive sprint swimmers (aged 19.7 years) showed much lower mean body height (168.3 cm), higher body mass (63.8 kg), and higher BF% (25%) compared to our results. Moreover, the same study suggested that BF% is a predictor of swimming performance in women [64]. Conversely, a more recent study of 90 female teenage national level swimmers (aged 13 to 19 years) suggested lower average BMI (19.0 kg/m^2^) and BF% (18.2%) compared to our results [65]. These apparently divergent results may be reconciled by age differences or preparedness and competition type and level. One small cross-sectional study on 9 US female collegiate swimmers and divers (body height: 173.8 cm and body mass: 65.3 kg), for instance, suggested that BF% is significantly higher pre-season, and the change is more evident in distance swimmers than sprinters [66]. Hence, anthropometry of elite-level female swimmers is subject to ongoing research and debate.

### 4.3. Dietary Intake and Serum Micronutrient Status

In our study, gymnasts had lower intake of energy and all macro- and micronutrients compared to swimmers. More importantly, dietary patterns of both indoor athlete groups had ample room for improvement. Gymnasts had below recommended energy intake [41], whereas both gymnasts and swimmers had below recommended intakes of fibre and PUFA [41]. In addition, gymnasts had below recommended intake of protein and 11 out of 13 micronutrients (except of vitamin B_12_ and zinc), whereas swimmers had below recommended intakes of vitamin D, calcium, potassium, and selenium [41, 42]. Conversely, intake of free sugars exceeded the SACN [44] and ESPGAHN [45] upper limit of <5% of daily energy intake in both groups (65 g/day, 17% of energy in gymnasts and 94 g/day, and 17% of energy in swimmers). Furthermore, intake of SFA also exceeded the upper limit in both groups.

Energy availability of 23 kcal/kg FFM/day of the gymnasts was even below the lower threshold recommendation (i.e., 30 kcal/kg FFM/day) [40]. This value has been cited as the threshold between normal and abnormal function based on disruption of luteinizing hormone (LH) pulsatility in women [67]. Irregular menstrual cycle may have a multifactorial etiology; however, the main reason that is well described in sport science literature is the reduced energy intake, especially with regards to low EA [68, 69]. Furthermore, the estimated carbohydrate intake of gymnasts and swimmers (3.3 and 5.1 g/kg BM/day) was barely suitable for low-intensity or skilled-based activity needs because 8–12 g/kg BM/day is recommended for > 4–5 h/day of moderate-to-high intensity exercise [40]. Percentages of total fat intake were in the upper recommended range for both groups (40 and 38% of daily energy intake, respectively, for gymnasts and swimmers vs. the recommended 30–40% [40]). Composition of fat was characterized by excessive intake of SFA (30 g/day, 18% of energy in gymnasts and 43 g/day, and 17% of energy in swimmers vs. recommended <10% of energy intake [41]), appropriate intake of MUFA in gymnasts and swimmers (14% and 13% of energy vs. the recommended ≥10 of energy), whereas low intake of PUFA was found in both groups (4% for both groups vs. the recommended 7–10%). Protein intake contributed 14% (or 1.0 g/kg BM/day) and 13% (or 1.2 g/kg BM/day) of energy in gymnasts and swimmers, respectively. Gymnasts consumed significantly lower amount of protein (1.0 vs. the threshold of 1.2 g/kg BM/day, *p*=0.002). Furthermore, fibre intake was markedly low for gymnasts and swimmers (11 and 17 g/day vs. the recommended ≥30 g/day [41]). Water intake of athletes needs to be determined individually [40] and cannot be interpreted in terms of achieving the recommendation. However, we assume that total water intake in both groups was lower than expected, especially due to low intake of fruits and vegetables, which both have high water content, and partly due to their intake of sports drinks containing carbohydrates with gelling properties (e.g., additionally limiting water intake) for swimmers.

Apart from being an important determinant of health status and anthropometry measures, dietary intake was found to be directly associated with effectiveness of training, performance, and recovery status among athletes [70, 71]. Therefore, dietary intake among athletes must be nutritionally adequate while at the same time limiting the intake of unfavourable nutrients, such as free sugars, sodium, trans-fatty acids, SFA, and dietary cholesterol [70]. In brief, we found that dietary intake, especially micronutrients, among participants in our study was suboptimal. This finding might be explained by the low total energy intake, especially in gymnasts, and their food choices characterized by very low intake of whole grains, beans, fruits, and vegetables and high intake of processed and ultraprocessed foods high in free sugars, salt, SFA, and trans-fatty acids.

Furthermore, we measured vitamin D intake from diet and supplements. It is well-known that it is hard to reach acceptable serum levels of vitamin D through diet alone [48], and supplementation above 40° latitude (which is the case in Slovenia) is advised during winter months (October to April) when the production of vitamin D through sun exposure is insufficient [41, 72]. Three gymnasts consumed vitamin D supplements every day, whereas none of the swimmers consumed vitamin D supplements. The 25 (OH) D values of the three gymnasts that consumed vitamin D supplements were well above the 100 nmol/L cutoff (one was even 172.2 nmol/L). Only one gymnast did not consume vitamin D supplements but had sufficient 25 (OH) D nonetheless. However, she reportedly took a 14-day training “vacation” at a lower geographical latitude (34° N) three months prior to the study.

It is also important to note that the frequency of daily consumption of food groups of vegetable origin were very limited, whereas the consumption of very important and healthy food groups (e.g., legumes, nuts and seeds, and fish) was alarmingly low in both groups of athletes. Together, an increase in these factors may greatly contribute to sufficient consumption of protein (in gymnasts), fibre, omega-3 PUFA, and numerous micronutrients [73].

The issue of inadequate dietary intake in artistic gymnasts is not novel. Several previous studies suggested inadequate intake of energy and micronutrients in artistic gymnasts on national teams [9, 71]. Our results, however, may partly be compared to only a handful of previous studies. One study in 33 US national team artistic female gymnasts reported an energy intake of 1678 kcal/day, which is 11% more than in our study. The study results also reported lower fat (18% vs. 40%) and higher carbohydrate (66% vs. 47%) intake than in our study. The researchers also found vitamin sufficiency (except for vitamin E), whereas minerals, such as calcium, zinc, and magnesium, were below reference values [71]. Two decades ago, Spanish researchers reported disturbing results in a sample of young elite-level female gymnasts (15.4 years, 161.3 cm, 42.4 kg on average) [9]. Briefly, the average energy intake was 1267 kcal, with a carbohydrate intake of 155.8 g (3.67 g/kg BM or 46.6% of energy). Additionally, the fat intake of Spanish gymnasts was lower than that among our gymnasts, both in relative (27.6% and 40%, respectively) and absolute terms (39.2 g and 67 g for Spanish and Slovenian gymnasts, respectively). Collectively, the hypocaloric diet of Spanish gymnasts resulted in extremely low BMI (16.3 kg/m^2^), and the authors suggested that their study results represented evidence of malnutrition [9]. In a rare recent investigation, the authors reported two case studies of one female artistic gymnast (*n* = 1, age = 18.5 years) and one rhythmic gymnast (*n* = 1; age: 16.1 years) who were both members of the Greek National Team [13]. Using a 7-day weighed food record protocol, the researchers found that only vitamin C, vitamin B_6_, and zinc exceeded daily recommended amounts, whereas calcium intake was insufficient and had the highest deviation from the recommended daily amount. Despite the fact that our results are hardly comparable to values reported in this case study, we can clearly see a similar trend. In brief, in both investigations, there was evident low energy intake (1712 kcal and 1514 kcal) and very low dietary fibre intake (14.7 vs. 11 g in Greek and our study). Conversely, the Greek study revealed much better micronutrient intake, which is probably a result of significantly higher energy intake resulting in increased intake of some micronutrients.

According to the available literature, dietary intake among swimmers is generally less problematic. However, a study with 85 US female collegiate swimmers suggests that the mean proportion of macronutrients was 25–30% energy from fat, 55–65% energy from carbohydrate, and 11–15% of energy from protein [74], which is different than in our study (38%, 54%, and 13%, respectively). The same study also reported a mean total energy intake of 3229 kcal/day, which is substantially higher (by 43%) than our results. However, energy needs depend on several factors, including type of swim stroke, body mass of the athlete, duration of activity, and seasonality (training vs. competing) [74]. In our study, 86% of swimmers competed in short-distance disciplines and participated in the study (as gymnasts) during the first competition period. Therefore, the lower energy intake may still be appropriate.

All serum micronutrients, both in gymnasts and swimmers, were within reference ranges, except for 25 (OH) D for both groups and S–P for gymnasts. Insufficient or deficient levels of 25 (OH) D were found in 77% of gymnasts and 57% of swimmers, with 42% of gymnasts and 21% of swimmers being outright deficient. However, this study was intentionally conducted just after the winter season, when serum levels of 25 (OH) D are expected to be naturally lowest in that specific period and at that geographic latitude [75]. Of note, only 53% of gymnasts and 79% of swimmers had S-vit B_12_ above the reference value of 258 pmol/L [46].

### 4.4. Cardiovascular Health

Our results suggest relatively favourable cardiovascular health status of included athletes both in terms of CVD risk profile [49, 50, 53] and safety factors [54–56]. However, some noteworthy differences, especially in blood lipids and blood pressure, between gymnasts and swimmers merit addressing.

Gymnasts had a less favourable lipid profile, higher LDL cholesterol, and lower HDL cholesterol levels, whereas swimmers had, on average, higher systolic BP. Almost two-thirds of gymnasts had LDL cholesterol levels above 2.6 mmol/L (41% above 3.0 mmol/L), which is higher than the currently recommended levels for long-term preservation of cardiovascular health [51, 52]. All gymnasts, however, had blood pressure within recommended values. Conversely, only one swimmer in five had LDL cholesterol levels above 2.6 mmol/L (7% above 3 mmol/L), but 36% of them had systolic blood pressure above the recommended values [53].

There are several possible explanations for these observed differences. Higher LDL cholesterol in gymnasts could be associated with dietary patterns, including lower energy intake, higher overall total fat, SFA, and free sugar intake and lower fibre intake. SFA and refined carbohydrates are associated with increased risk of CVD [21, 22], whereas dietary fibre yields a reduction in LDL cholesterol (via reduced gastrointestinal absorption) [76]. Notably, the researchers in a study on 40 Danish and Swedish female national teams and competitive clubs found an association between the reduced energy availability and higher LDL cholesterol [77]. Lower systolic BP in gymnasts, on the other hand, may be partially explained by lower energy intake and the fact that 35% of gymnasts systematically took magnesium supplements since a calorie-restricted diet and magnesium supplementation are associated with reduced BP [78, 79].

Higher systolic BP in swimmers can also be explained by dietary patterns. Swimmers' relative intake (per kg BM/day) analysis showed that they consumed more energy, carbohydrates, sugar, and total fat than gymnasts. Importantly, four times more swimmers consumed meat daily. In comparison, 47% of gymnasts consumed a vegetarian or sometimes vegetarian diet, while 86% of swimmers characterized their diet as omnivorous. Vegetarian diets, if properly designed, have significantly favourable CVD risk [80]. Importantly, three prospective United States of America (US) cohort studies suggested that a healthier vegetarian diet was found to be associated with lower CVD risk, whereas a less healthy diet was found to be associated with higher CVD risk [81]. Importantly, heterogeneity in dietary patterns among gymnasts and swimmers in a relatively small sample may also be associated with observed results.

A rare, older cross-sectional study on Portuguese gymnasts (albeit rhythmic gymnasts) [82] and a cross-sectional study on US swimmers showed lower lipid status among their gymnasts compared with ours and a similar BP pattern among their swimmers compared with ours [83]. Specifically, a study on 20 adolescent female rhythmic gymnasts from the Portuguese national team showed an average S-cholesterol of 4.2 mmol/L (4.7 mmol/L in our study), an LDL cholesterol of 2.3 mmol/L (2.7 mmol/L in our study), and an HDL cholesterol of 1.6 mmol/L (1.5 mmol/L in our study) [82]. The study on middle-age US swimmers of both genders (17 men and 8 woman) found a similar average BP value of 128/74 mmHg compared with the BP for our swimmers (126/73 mmHg in our study) [83]. We assume that these differences in terms of LDL cholesterol between gymnasts and systolic BP between swimmers might be partly due to different dietary intakes and population differences.

In terms of safety factors, all athletes had S-glucose, S-UA, and haemoglobin within recommendations [54–56], except one gymnast who had haemoglobin bellow 120 g/L (114 g/L). In brief, according to a recent systematic review, to avoid increased risk of all-cause mortality, including cardiovascular events and stroke, researchers suggest maintaining S-glucose between 4 and <4.6 mmol/L [84]. However, our gymnasts and swimmers had S-glucose 4.3 and 4.9 mmol/L, respectively. Therefore, in the long-term, it would be advisable for swimmers to further improve their S-glucose values. Furthermore, there is no clear consensus about the normal S-UA reference range. However, there is a known association between S-UA and health risk that is biphasic [29]. Specifically, in a large cohort study, researchers found a U-shaped association. Levels between 300 and 410 *μ*mol/L (our gymnasts and swimmers had S-UA 265 and 282 *μ*mol/L) were shown to be associated with the lowest mortality [85].

### 4.5. Strengths and Limitations

We included all female members of the Slovenian gymnast and swimming teams, representing two comprehensive age-matched, elite-level indoor sports populations rather than samples. In fact, none of the invited gymnasts and swimmers declined to participate, and none were excluded from the study. No missing data for study outcome were presented. Furthermore, the study design was comprehensive with data acquisition completed within three days, thus minimising seasonal confounders.

The study has some limitations inherent to the sample size. The sample size was relatively small, and therefore, the results should be interpreted with caution and should ideally be replicated in larger samples. In addition, we acknowledge the limitations of collecting data using an FFQ. Initially, we planned to evaluate the dietary intake also by a three-day weighted dietary protocol, which is a golden standard for evaluating the dietary intake. However, the coaches of the participating elite-level artistic gymnasts and swimmers advised us against it, as it would be too time-consuming for the participants. However, the FFQ allowed us to distinguish between different dietary patterns in subpopulations [36, 86]. The limited number of items in the FFQ and the one-year recall period may have weakened the discriminative power of FFQs, but our athletes, based on introductory interviews with coaches and athletes, had a relatively stable dietary pattern. Importantly, the relative validity of the used FFQ was previously compared with 7-day estimated diet records, and the results suggested that this FFQ is reasonably valid in both genders and across different age categories for most food groups. However, poor ranking agreement was found for some food groups (e.g., bread and cereals, potatoes, and grains) [38]. FFQ was validated in Dutch population but not in Slovenian population. Of note, existing literature also demonstrates substantial variability between dietary assessment methods with under- and misreporting of dietary intake [87, 88]. This issue is especially relevant for female artistic gymnasts because of known problems with under-reporting of energy intake by elite-level female gymnasts [88]. Last, blood assays were obtained from two national medical laboratories separately for gymnasts and swimmers that may represent a potential limitation for the results obtained.

## 5. Conclusion

Gymnasts in our study were shorter and lighter and had higher BMI, lower FFM, and comparable BF% to swimmers. Energy availability, especially in gymnasts, was too low. Both groups consumed too much of free sugars, total fat, and SFA and low intake of proteins (gymnasts only), polyunsaturated fatty acids, fibre, and several micronutrients (11/13 in gymnasts and 4/13 in swimmers). Both groups, especially gymnasts, have room for dietary optimisation, which would include increasing the intake of whole plant-based foods, especially whole grains, legumes, fresh fruits, and vegetables as well as small sea fish (sardines, sardines, and anchovies), while decreasing the intake of processed, ultraprocessed, and fried foods. Dietary optimisation would also benefit their training and athletic performance.

In gymnasts and swimmers, the most challenging serum micronutrient shortcomings were 25 (OH) D and S-vit B_12_ levels. As 25 (OH) D and S-vit B_12_ were low (25 (OH) D: for 77% of gymnasts and 57% of swimmers; S-vit B_12_: for 47% of gymnasts and 21% of swimmers), it should be regularly tested and supplemented in those with suboptimal levels. CVD risk and safety factors were mostly within guideline-recommended ranges. However, gymnasts had more unfavourable lipid profiles (increased LDL cholesterol), whereas swimmers had more unfavourable BP profiles (increased systolic BP).

Despite the novelty of our results, single assessment does not allow for a definitive conclusion concerning anthropometry or dietary and cardiovascular health. For more conclusive data, frequent comprehensive analysis of same female gymnasts is warranted.

## Figures and Tables

**Table 1:** Characteristics of gymnasts and swimmers.

Variable	Gymnasts (*n* = 17)	Swimmers (*n* = 14)	*p* value
Body composition^‡^			
Height (cm)	159.8 ± 6.2	173.0 ± 5.5	<0.001
Body mass (kg)	54.8 ± 5.3	60.4 ± 7.0	0.057
BMI (kg/m^2^)	21.5 ± 3.8	20.1 ± 1.9	**0.043**
BF (%)	22.5 ± 3.8	22.8 ± 3.7	0.889
BF (kg)	12.4 ± 2.7	13.8 ± 3.1	0.578
FFM (kg)	42.4 ± 3.7	46.6 ± 5.5	**0.024**
Menstruation status			
Mean age of the menstruation (years)^‡^	13.9 ± 1.4	13.4 ± 1.4	
Regular cycle (*n* (%))	14 (82)	11 (79)	
Age of the first menstruation (years)			
≤11	1	0	
11 ≤ 12	2	4	
12 ≤ 13	0	4	
13 ≤ 14	8	4	
14 ≤ 15	3	1	
17	1	1	
not yet	2	0	
Dietary pattern (*n* (%))			
Omnivores	9 (53)	12 (86)	
Vegetarian or occasionally vegetarian	8 (47)	2 (14)	

^‡^Data are presented as the mean (standard deviation). A Mann–Whitney *U* test was applied to compare differences between groups. Statistically significant *p* values are presented in bold. ^†^BF is body fat expressed in percentage (%) and in kilograms (kg).^††^FFM is fat free mass.

**Table 2:** Dietary intake among elite-level artistic gymnasts and swimmers gymnasts and swimmers compared with recommendations [40–45].

Variable (/day)	Recommendations	Gymnasts	Swimmers	*p* value
*G* vs. recommendations/S vs. Recommendations				
Energy availability (kcal/kg FFM)^EA^	45	23 ± 3	33 ± 10	**<0.001**/**<0.05**
Macronutrients				
Carbohydrates (g/kg BM)	8–12	3.3 ± 0.8	5.1 ± 1.6	**<0.001**/**<0.001**
Free sugars (% E)^FS^	<5	17 ± 5	17 ± 7	**0.001**/**0.001**
Dietary fibre (g)	≥30	11 ± 2	17 ± 3	**0.001**/**0.001**
Total fat (% E)	30–40	40 ± 13	38 ± 13	0.159/0.286
SFA (% E)	≤10	18 ± 7	17 ± 7	**<0.001**/**<0.002**
MUFA (% E)	≥10	14 ± 4	13 ± 3	**0.001**/**0.005**
PUFA (% E)	7–10	4 ± 1.0	4 ± 1.0	**<0.001**/**<0.001**
Cholesterol (mg)	<300	148 ± 63	190 ± 90	**<0.001**/**<0.001**
Protein (g/kg BM)	1.2	1.0 ± 0.2	1.2 ± 0.2	**0.002**/0.583
Micronutrients				
Vitamins				
B_6_ (mg)	1.4	0.8 ± 0.3	1.8 ± 0.2	**<0.001**/0.463
B_12_ (*μ*g)	4	4.7 ± 2.9	10.5 ± 4.0	0.737/**<0.001**
C (mg)	90	30 ± 17	107 ± 69	**<0.001**/0.615
*D* (*μ*g)	20	5.5 ± 9.6	3.5 ± 3.5	**<0.001**/**0.001**
*E* (mg)	12	6.3 ± 2.4	15.7 ± 11.6	**<0.001**/0.583
Minerals				
Calcium (mg)	1200	629 ± 274	806 ± 228	**<0.001**/**<0.001**
Magnesium (mg)	350	292 ± 80	333 ± 79	**0.013**/0.286
Phosphorus (mg)	1250	924 ± 192	1236 ± 188	**<0.001**/0.463
Potassium (mg)	4000	1731 ± 475	2561 ± 373	**<0.001**/**<0.001**
Sodium (mg)	1500	901 ± 347	1576 ± 427	**<0.001**/0.761
Trace elements				
Iron (mg)	15	9 ± 4	14 ± 7	**<0.001**/0.263
Zinc (mg)	7	7 ± 2	9 ± 6	0.813/0.104
Selenium (mg)	60	30 ± 11	46 ± 16	**<0.001/0.007**

Data are presented as the mean (standard deviation). One-sample Wilcoxon signed-rank test was applied for comparison against recommendations. G vs. recommendations average values for gymnasts compared with recommendations; S vs. recommendations average values for swimmers compared with recommendations. Statistically significant p values are presented in bold. ^EA^Energy availability (EA) was calculated from energy intake (EI), exercise energy expenditure (EEE), and FFM. Exercise energy expenditure were reported by coaches from past measurements (i.e., using heart rate monitoring) during similar competition circumstances and only for few of Slovenia's best gymnasts (average: 514 ± 72 kcal) and swimmers (average: 723 ± 40 kcal). ^FS^Free sugars: all monosaccharides and disaccharides added to foods and beverages by the manufacturer, cook, or consumer (e.g., added sugars) plus that naturally present in honey, syrups, fruit juices, and fruit juice concentrates (defined by the World Health Organization (WHO) [43], adapted by SACN [44] and ESPGHAN [45]). SFA saturated fatty acids, MUFA monounsaturated fatty acids, PUFA polyunsaturated fatty acids. Atwater energy conversion factors were used (kcal/g): carbohydrates and protein 4, dietary fibre 2, fat 9 [60].

**Table 3:** Serum micronutrient status of elite-level artistic gymnasts and swimmers.

Parameter	Reference	Gymnasts	Swimmers	*p* value∗
Vitamins				
S-vit B_12_ (pmol/L)^†^	≥258^†^	292 ± 86	339 ± 93	0.204
25 (OH) D (nmol/L)^††^	≥75^††^	65 ± 36	55 ± 42	0.634
Minerals‡				
S-Ca (mmol/L)	2.10–2.60	2.41 ± 0.06	2.45 ± 0.06	0.330
S-Mg mmol/L)	0.60–1.10	0.87 ± 0.50	0.83 ± 0.56	0.079
S-P (mmol/L)	0.84–1.45	1.46 ± 0.17	1.28 ± 0.20	**0.018**
S-K (mmol/L)	3.8–5.5	4.82 ± 0.27	4.47 ± 0.30	**0.004**
Trace element‡				
S-Fe (*μ*mol/L)	10.7–28.6	16 ± 7	21 ± 7	0.052

Data are means (standard deviation). A Mann‐Whitney U test was applied to compare differences between groups. ^∗^*p* value represents group comparisons (gymnasts vs. swimmers). Statistically significant p values are written in bold.^ ‡^Serum vitamin B_12_ (S-vit B_12_ B_12_) was compared with value suggested by German researchers to prevent neurocognitive disorders late in life [46]. ^††^For further analysis of 25 (OH) D status, we used three categories (e.g., sufficiency: >75 nmol/L, insufficiency: 50–≤75 nmol/L, and deficiency:50 nmol/L) [48]. ^‡^Concentrations of serum minerals and trace elements were compared with reference values from the University Medical Centre Ljubljana, Slovenia, the national core laboratory [49].

**Table 4:** Cardiovascular (CV) health and safety factors compared among elite-level artistic gymnasts and swimmers.

Parameter	Recomm./refer.^^^	Gymnasts	Swimmers	*p* value
S-cholesterol (mmol/L)^†^	<5.2	4.7 ± 0.7	4.3 ± 0.6	0.196
LDL cholesterol (mmol/L)^††^	<3.4	2.7 ± 0.5	2.2 ± 0.5	**0.011**
HDL cholesterol (mmol/L)^†^	>1.3	1.5 ± 0.2	1.8 ± 0.3	**<0.001**
Triglycerides (mmol/L)^††^	<1.7	0.8 ± 0.2	0.8 ± 0.3	0.617
Blood pressure (mmHg)^†††^				
Systolic	≤129	107 ± 9	126 ± 9	**<0.001**
Diastolic	≤84	69 ± 7	73 ± 8	0.175
S-glucose (mmol/L)^‡^	<5.8	4.3 ± 0.3	4.9 ± 0.5	**0.002**
S-UA (*μ*mol/L)^‡‡^	<360	265 ± 60	282 ± 52	0.500
Haemoglobin (g/L)^‡‡‡^	≥120	137 ± 9	136 ± 6	0.450

Data are presented as the means (standard deviation). A Mann‐Whitney U test was applied to compare differences between groups. Statistically significant *p* values are written in bold. ^Recommendations or reference values:†S-cholesterol and HDL cholesterol reference values from University Medical Centre Ljubljana, Slovenia, the national core laboratory [49].^††^Low-density lipoprotein cholesterol (LDL cholesterol) and triglycerides recommendation used are from the European Atherosclerosis Society (EAS) [50].^†††^BP recommendations from the European Society of Cardiology (ESC) were used [53]. ^‡^S-glucose recommendations were from the European Diabetes Epidemiology Group for lean adults (BMI 25 kg/m^2^) [55]. ^‡‡^Serum uric acid (S-UA) consensual threshold used for all healthy subjects [54]. ^‡‡‡^For haemoglobin, we used recommended cutoffs for a nonanaemic state from the World Health Organization (WHO) for nonpregnant females (>120 g/L) [56].

## Data Availability

The data used to support the findings of this study are included within the article.

## Supplementary Materials

Supplementary Materials
Table 1S. Dietary intake from foods and supplements among elite-level artistic gymnasts and swimmers. Table 2S. Dietary intake status among elite-level artistic gymnasts and swimmers per kcal/FFM/day (for energy) and per kg BM/day (for macro- and micronutrients).
