# Supplementary material for: Nutritional Status and Cardiovascular Health in Female Adolescent Elite-Level Artistic Gymnasts and Swimmers: A Cross-Sectional Study of 31 Athletes
Source: J Nutr Metab. 2021 Jan 12;2021:8810548. doi: 10.1155/2021/8810548 (PMC7815399; doi:10.1155/2021/8810548)
Supplement: Supplementary Materials — Table 1S. Dietary intake from foods and supplements among elite-level artistic gymnasts and swimmers. Table 2S. Dietary intake status among elite-level artistic gymnasts and swimmers per kcal/FFM/day (for energy) and per kg BM/day (for macro- and micronutrients). [file 8810548.f1.zip › 8810548.f1/TABLE 1S (3).docx]

TABLE 1S: Dietary intake from foods and supplements among elite-level artistic gymnasts and swimmers.

| Variable | Gymnasts (n=17) | Swimmers (n=14) | *p* - value |
| --- | --- | --- | --- |
| Energy intake (kcal/day) | 1514 ± 258 | 2263 ± 407 | **< 0.001** |
| **Macronutrients (per day)** |  |  |  |
| Carbohydrates (g) | 177 ± 39 | 305 ± 72 | **< 0.001** |
| (% E) | 47 ± 11 | 54 ± 9 | **0.013** |
| Total sugars (g)^TS^ | 110 ± 31 | 189 ± 50 | **< 0.001** |
| Free sugars (g)^FS^ | 65 ± 25 | 94 ± 45 | **0.021** |
| (% E) | 17 ± 5 | 17 ± 7 | 0.721 |
| Starches (g) | 66 ± 34 | 95 ± 39 | **0.039** |
| Dietary fibre (g) | 11 ± 2 | 17 ± 3 | **< 0.001** |
| Total fat (g) | 67 ± 24 | 95 ± 32 | **0.018** |
| (% E) | 40 ± 13 | 38 ± 13 | 0.521 |
| SFA (g) | 30 ± 13 | 43 ± 19 | 0.062 |
| (% E) | 18 ± 7 | 17 ± 7 | 0.765 |
| MUFA (g) | 23 ± 8 | 33 ± 11 | **0.005** |
| (% E) | 14 ± 4 | 13 ± 3 | 0.405 |
| PUFA (g) | 7 ± 2 | 10 ± 3 | **0.002** |
| (% E) | 4 ± 1 | 4 ± 1 | 0.781 |
| Cholesterol (mg) | 148 ± 63 | 190 ± 90 | 0.159 |
| Protein (g) | 49 ± 12 | 67 ± 10 | **< 0.001** |
| (% E) | 14 ± 3 | 13 ± 2 | 0.246 |
| Water (l)^TW^ | 1.97 ± 0.21 | 2.54 ± 0.27 | **< 0.001** |
| **Micronutrients (per day)** |  |  |  |
| **Vitamins** |  |  |  |
| B_6_ (mg) | 0.8 ± 0.3 | 1.8 ± 0.2 | **< 0.001** |
| B_12_ (µg) | 4.7 ± 2.9 | 10.5 ± 4.0 | **0.003** |
| C (mg) | 30 ± 17 | 107 ± 69 | **< 0.001** |
| D (µg) | 5.5 ± 9.6 | 3.5 ± 3.5 | 0.551 |
| E (mg) | 6.3 ± 2.4 | 15.7 ± 11.6 | **0.002** |
| **Minerals** |  |  |  |
| Calcium (mg) | 629 ± 274 | 806 ± 228 | 0.122 |
| Magnesium (mg) | 292 ± 80 | 333 ± 79 | 0.184 |
| Phosphorus (mg) | 924 ± 192 | 1236 ± 188 | **< 0.001** |
| Potassium (mg) | 1731 ± 475 | 2561 ± 373 | **< 0.001** |
| Sodium (mg) | 901 ± 347 | 1576 ± 427 | **< 0.001** |
| **Trace elements** |  |  |  |
| Iron (mg) | 9 ± 4 | 14 ± 7 | **0.017** |
| Zinc (mg) | 7 ± 2 | 9 ± 6 | **0.019** |
| Selenium (mg) | 30 ± 11 | 46 ± 16 | **0.004** |

Data are presented as the means (standard deviation). A Mann-Whitney U test was applied to compare differences between groups. Statistically significant *p* - values (<0.05) are presented in bold. ^TS^Total sugars: all monosaccharides and disaccharides; free sugars^FS^ plus sugars naturally present in foods (e.g., lactose in milk, fructose in fruits) [43]. ^FS^Free sugars: all monosaccharides and disaccharides added to foods and beverages by the manufacturer, cook or consumer (e.g., added sugars) plus that naturally present in honey, syrups, fruit juices and fruit juice concentrates (defined by World Health Organization (WHO) [43], adapted by SACN [44] and ESPGHAN [45]. SFA = saturated fatty acids, MUFA = monounsaturated fatty acids, PUFA = polyunsaturated fatty acids. Atwater energy conversion factors were used (kcal/g): carbohydrates and protein = 4, dietary fibre = 2, fat = 9 [60]. ^TW^Total water from solid foods and beverages (including sports drinks).
