# Supplementary material for: Nutritional Status and Cardiovascular Health in Female Adolescent Elite-Level Artistic Gymnasts and Swimmers: A Cross-Sectional Study of 31 Athletes
Source: J Nutr Metab. 2021 Jan 12;2021:8810548. doi: 10.1155/2021/8810548 (PMC7815399; doi:10.1155/2021/8810548)
Supplement: Supplementary Materials — Table 1S. Dietary intake from foods and supplements among elite-level artistic gymnasts and swimmers. Table 2S. Dietary intake status among elite-level artistic gymnasts and swimmers per kcal/FFM/day (for energy) and per kg BM/day (for macro- and micronutrients). [file 8810548.f1.zip › 8810548.f1/TABLE 2S (3).docx]

TABLE 2S: Dietary intake status among elite-level artistic gymnasts and swimmers per kcal/FFM/day (for energy) and per kg BM/day (for macro- and micronutrients).

| Variable | Unit | Gymnasts | Swimmers | *p* - value |
| --- | --- | --- | --- | --- |
| Energy intake | kcal/kg FFM/day | 36 ± 7 | 49 ± 10 | **0.001** |
| **Macronutrients** |  |  |  |  |
| Carbohydrate | g/kg BM/day | 3.3 ± 0.8 | 5.2 ± 1.6 | **< 0.001** |
| Total sugar | g/kg BM/day | 2.0 ± 0.7 | 3.2 ± 1.1 | **0.003** |
| Free sugar | g/kg BM/day | 1.3 ± 0.5 | 1.8 ± 0.8 | 0.052 |
| Starches | g/kg BM/day | 1.2 ± 0.6 | 1.6 ± 0.6 | 0.112 |
| Dietary fiber | g/kg BM/day | 0.21 ± 0.06 | 0.30 ± 0.07 | **< 0.001** |
| Total fat | g/kg BM/day | 1.2 ± 0.5 | 1.6 ± 0.6 | **0.043** |
| SFA | g/kg BM/day | 0.57 ± 0.26 | 0.73 ± 0.33 | 0.122 |
| MUFA | g/kg BM/day | 0.43 ± 0.17 | 0.55 ± 0.19 | 0.074 |
| PUFA | g/kg BM/day | 0.12 ± 0.04 | 0.16 ± 0.04 | **0.032** |
| Cholesterol | mg/kg BM/day | 2.7 ± 1.2 | 3.2 ± 1.5 | 0.361 |
| Protein | g/kg BM/day | 1.0 ± 0.2 | 1.2 ± 0.2 | **0.009** |
| **Micronutrients** |  |  |  |  |
| **Vitamins** |  |  |  |  |
| Vitamin B_6_ | mg/kg BM/day | 0.014 ± 0.005 | 0.030 ± 0.02 | **< 0.001** |
| Vitamin B_12_ | µg/kg BM/day | 0.09 ± 0.06 | 0.17 ± 0.13 | **0.005** |
| Vitamin C | mg/kg BM/day | 0.55 ± 0.54 | 1.8 ± 1.2 | **< 0.001** |
| Vitamin D | µg/kg BM/day | 0.1 ± 0.2 | 0.06 ± 0.05 | 0.525 |
| Vitamin E | mg/kg BM/day | 0.12 ± 0.05 | 0.26 ± 0.18 | **0.012** |
| **Minerals** |  |  |  |  |
| Calcium | mg/kg BM/day | 11.6 ± 5.0 | 13.5 ± 3.7 | 0.177 |
| Magnesium | mg/kg BM/day | 5.4 ± 1.7 | 5.6 ± 1.2 | 0.843 |
| Phosphorus | mg/kg BM/day | 17 ± 4 | 21 ± 4 | **0.012** |
| Potassium | mg/kg BM/day | 32 ± 10 | 43 ± 7 | **0.003** |
| Sodium | mg/kg BM/day | 16.6 ± 6.4 | 26.0 ± 5.3 | **< 0.001** |
| **Trace elements** |  |  |  |  |
| Iron | mg/kg BM/day | 0.17 ± 0.08 | 0.23 ± 0.10 | 0.112 |
| Zinc | mg/kg BM/day | 0.12 ± 0.04 | 0.16 ± 0.07 | 0.142 |
| Selenium | mg/kg BM/day | 0.56 ± 0.20 | 0.76 ± 0.23 | **0.019** |

Data are presented as the means (standard deviation). A Mann-Whitney U test was applied to compare differences between groups. Statistically significant *p* - values are presented in bold.
